# Harmonizing community-based health worker programs for HIV: a narrative review and analytic framework

**DOI:** 10.1186/s12960-017-0219-y

**Published:** 2017-07-03

**Authors:** Jan-Walter De Neve, Chantelle Boudreaux, Roopan Gill, Pascal Geldsetzer, Maria Vaikath, Till Bärnighausen, Thomas J. Bossert

**Affiliations:** 1 Institute of Public Health, Heidelberg University, Heidelberg, 69120 Germany; 2 Department of Global Health and Population, Harvard T.H. Chan School of Public Health, 665 Huntington Avenue, Boston, MA 02115 United States of America; 3 BC Women’s Hospital, University of British Columbia, 4500 Oak St, Vancouver, Canada; 4 Africa Health Research Institute, Mtubatuba, KwaZulu-Natal South Africa

**Keywords:** Community health workers, Harmonization, Low- and middle-income countries, HIV

## Abstract

**Background:**

Many countries have created community-based health worker (CHW) programs for HIV. In most of these countries, several national and non-governmental initiatives have been implemented raising questions of how well these different approaches address the health problems and use health resources in a compatible way. While these questions have led to a general policy initiative to promote harmonization across programs, there is a need for countries to develop a more coherent and organized approach to CHW programs and to generate evidence about the most efficient and effective strategies to ensure their optimal, sustained performance.

**Methods:**

We conducted a narrative review of the existing published and gray literature on the harmonization of CHW programs. We searched for and noted evidence on definitions, models, and/or frameworks of harmonization; theoretical arguments or hypotheses about the effects of CHW program fragmentation; and empirical evidence. Based on this evidence, we defined harmonization, introduced three priority areas for harmonization, and identified a conceptual framework for analyzing harmonization of CHW programs that can be used to support their expanding role in HIV service delivery. We identified and described the major issues and relationships surrounding the harmonization of CHW programs, including key characteristics, facilitators, and barriers for each of the priority areas of harmonization, and used our analytic framework to map overarching findings. We apply this approach of CHW programs supporting HIV services across four countries in Southern Africa in a separate article.

**Results:**

There is a large number and immense diversity of CHW programs for HIV. This includes integration of HIV components into countries’ existing national programs along with the development of multiple, stand-alone CHW programs. We defined (i) coordination among stakeholders, (ii) integration into the broader health system, and (iii) assurance of a CHW program’s sustainability to be priority areas of harmonization. While harmonization is likely a complex political process, with in many cases incremental steps toward improvement, a wide range of facilitators are available to decision-makers. These can be categorized using an analytic framework assessing the (i) health issue, (ii) intervention itself, (iii) stakeholders, (iv) health system, and (v) broad context.

**Conclusions:**

There is a need to address fragmentation of CHW programs to advance and sustain CHW roles and responsibilities for HIV. This study provides a narrative review and analytic framework to understand the process by which harmonization of CHW programs might be achieved and to test the assumption that harmonization is needed to improve CHW performance.

**Electronic supplementary material:**

The online version of this article (doi:10.1186/s12960-017-0219-y) contains supplementary material, which is available to authorized users.

## Background

As the HIV epidemic matures, the emphasis of community-based health worker (CHW) programs is transitioning to long-term HIV services, and the associated health system demands have grown [1]. With health workforce constraints, the World Health Organization (WHO) has promoted “task shifting” toward less costly and more available health personnel [2]. One implication of this has been the rapid expansion of both national and donor-driven/supported CHW programs, which has occurred in parallel with a shift in CHW responsibilities to include activities beyond health promotion. There is an increasing expectation that CHWs will participate in disease surveillance and data collection activities, as well as play an active role in the diagnosis and referral for care, and in efforts to roll out anti-retroviral treatment (ART) coverage [3]. CHW programs have been suggested to play a “transformative” role in scaling up HIV services for achieving the 90-90-90 treatment goals—including through community-level “test-and-treat” initiatives [4–9], differentiated care models [10], and there have been calls to dramatically increase the number of CHWs in HIV endemic settings [11–13].

However, as this transition to greater responsibility for CHWs has occurred, these cadres and the broader “community health system” have generally received increased scrutiny [14, 15]. Researchers and policy-makers have noted a number of challenges to the implementation of task shifting for HIV treatment and care, including the integration of CHWs into national health systems [3] and political and financial sustainability of CHW programs for HIV [16]. An important issue is the dynamic and utilization across national cadres and the donor-driven/supported cadres that were created for vertical programs such as HIV. As the resources aimed at (and reliance upon) these cadres increase, there is an increasing need to consider how to streamline CHW-led HIV activities to lend greater effectiveness and efficiencies [17]. In addition, heavy reliance on donor funding for many CHW cadres supporting HIV service delivery raises urgency for greater consideration of long-term sustainability [18–20].

A range of policy initiatives have increasingly promoted alignment of CHW initiatives [21–24]. In 2013, the Global Health Workforce Alliance (GHWA), an alliance of government leaders, donors, health workers, and civil society—facilitated by the United States Agency for International Development (USAID), the Norwegian Government’s agency for development (NORAD), and members of the Frontline Health Workers Coalition (FHWC)—announced their commitment for the need to “harmonize” support of community health workers [22, 25]. The announcement built on three guiding principles for the harmonization of CHW programs (the “three ones”): one national strategy, one authority, and one monitoring and accountability framework [22]. This public commitment signaled a broad argument for the need to develop a coherent and harmonized approach to community-based health worker support within countries. The CHW commitment helped bring critical attention to the harmonization of CHW programs for HIV because of the history of CHW cadre creation specific for HIV and expansion of cadre responsibilities with the push for achieving 90-90-90 goals [9]. Harmonization of CHW programs for HIV may have been further impeded by an absence of an understanding of key harmonization issues and history of development of programs under an emergency response for HIV. Few systematic efforts have been undertaken to understand the process by which harmonization of CHW programs might be achieved, and there is a lack of a common language and conceptual framework to inform future research efforts and policy (see Additional file 1 for additional background information on CHWs and CHW program fragmentation).

To address this gap in the literature, the narrative review and analytic framework in this study have been compiled to build upon these existing efforts and guide the development of country case studies to investigate factors for harmonization of community-based health workers for HIV in Lesotho, Mozambique, South Africa, and Swaziland (De Neve JW, Garrison-Desany H, Andrews KA, Sharara N, Boudreaux C, Gill R, Geldsetzer P, Vaikath M, Bärnighausen T, Bossert TJ. Harmonization of community health worker programs for HIV: a four-country qualitative study in Southern Africa, submitted). First, we further define the concept of harmonization, introduce three priority areas for harmonization (coordination, integration, and sustainability), and include an overview of factors thought to facilitate or hinder each. We then provide an analytic framework, first introduced by Atun et al. in 2010 [26], and incorporate the three priority areas in the framework.

## Methods

### Documents

We conducted a narrative review of the existing published and gray literature. We conducted multiple rounds of literature searches in PubMed and Google Scholar. The search strategy was conducted iteratively using English search terms, beginning with broad search terms (e.g., “fragmentation,” “community health worker programs”) and progressively expanded based on findings (e.g., “coordination,” “integration,” “sustainability”). We supplemented search results with several relevant publications previously known to the study team, including previous literature reviews [27–29] and seminal works [20, 30, 31] on the harmonization of health projects and programs. The search strategy also included manual searches of bibliographies of previous literature on health program-related harmonization. We searched articles regardless of date of publication. We first arbitrarily selected and reviewed the full-text versions of several articles from diverse fields with our search terms in the title to get a sense of the range of definitions and conceptions of the term in the social sciences. We then reviewed the first 100 titles of articles which included our search terms anywhere in the text. We reviewed the full-text versions of all articles whose primary focus was related to the harmonization of CHW programs. In total, we reviewed the full-text versions of approximately 50 articles, book chapters, and case studies. We prioritized articles for analysis that focused on issues related to harmonization of CHW programs and CHW-led HIV services in low- and middle-income settings. In our full-text review, we searched for and noted evidence on three categories: (i) definitions, models, and/or frameworks related to harmonization; (ii) theoretical arguments or hypotheses about the effects of CHW program fragmentation and/or harmonization; and (iii) empirical evidence.

### Analysis

Our analysis of documents proceeded in three steps. First, we further defined the concept of harmonization and identified three priority areas for the harmonization of CHW programs based on two previous articles related to harmonization. Specifically, we built on a previous multi-country study on the sustainability of donor-supported health projects by Bossert [31] and on a GHWA report on the coordination, integration, and sustainability of CHW programs [22]. These articles pioneered work on harmonization of donor-supported health programs and informed our identification and definition of priority areas for CHW program harmonization. In our synthesis of reviewed documents, we then described major issues and relationships surrounding these priority areas for harmonization of CHW programs, including key advantages, disadvantages, facilitators, and barriers for each of the three areas. We focused our synthesis on CHW-led delivery of HIV services such as HIV education, HIV testing campaigns, ART adherence counseling and monitoring, home-based care delivery, and community supply of ART. When evidence on HIV services specifically was scarce, we additionally aimed to describe evidence on CHW programs that offered related health services (such as sexual and reproductive health services [32]). Second, in the absence of a comprehensive framework for harmonization, we extended an existing framework for the integration of health services, previously suggested by Atun et al. [26], to our three priority areas of harmonization. Third, we used this analytic framework to map findings from our narrative review. As noted above, this analysis serves as a basis for a separate, empirical study in Southern Africa aimed at understanding how the harmonization of existing CHW programs supporting HIV might be achieved (De Neve JW, Garrison-Desany H, Andrews KA, Sharara N, Boudreaux C, Gill R, Geldsetzer P, Vaikath M, Bärnighausen T, Bossert TJ: Harmonization of community health worker programs for HIV: a four-country qualitative study in Southern Africa, submitted). Our emphasis has been to clearly define harmonization of CHW programs for HIV; to identify priority areas, a set of factors likely to facilitate or inhibit each; and to suggest an analytic framework that permits a systematic assessment of existing CHW programs for HIV.

## Results

### Definition and three priority areas for harmonization

Based on our review, we define “harmonization” broadly as public and non-state programs and initiatives that are compatible with larger health systems and the collaboration between all involved actors to contribute together to a comprehensive and sustainable systems approach in advocacy, programming, funding, implementing, monitoring, and building the knowledge base for CHW programs for HIV [22]. Harmonization of CHW programs for HIV can occur along a number of dimensions. We identified *coordination* among development partners and other stakeholders, *integration* into the broader health system, and assurance of a CHW program’s *sustainability* to be the priority areas (see Table 1 for an overview). Aid coordination is defined as “any activity or set of activities, formal or non-formal, at any level, undertaken by the recipient in conjunction with donors, individually or collectively, which ensures that foreign inputs to the health sector enable the health system to function more effectively…” [22]. Among CHW programs, coordination efforts seek to reduce duplication, fragmentation, confusion created by competing models, and overlap of responsibilities of differently trained CHWs in the same geographic areas [22, 26]. Integration is defined as “the extent, pattern, and rate of adoption and eventual assimilation of health interventions into each of the critical functions of a health system” [26]. Integration refers to the absorption of CHW programs into existing networks of larger health and/or community health systems, primarily the Ministries of Health or large private providers (non-governmental or commercial). Sustainability is defined as “the continued use of program components and activities for the continued achievement of desirable program and population outcomes” [18]. Sustainability is a key element for CHW programs supported by transitioning donor funding, such as PEPFAR [33, 34] or the Global Fund [35]. We explore in detail possible advantages and mediators of each of these priority areas of harmonization below.

**Table 1:** Three priority areas for harmonization of CHW programs

1. Coordination: Activities undertaken to ensure that inputs into the health sector enable the health system to function more effectively and in accordance with local priorities over time [61]. Among CHW programs, coordination efforts seek to reduce duplication, fragmentation, confusion created by competing models, and overlap of responsibilities of differently trained CHWs in the same geographic areas [22, 26].	
2. Integration: Absorption of CHW programs into existing networks of larger health systems such as the Ministries of Health or large private providers. Integration is defined as the extent, pattern, and rate of adoption and eventual assimilation of health interventions into each of the critical functions of the health system [26].	
3. Sustainability: Continued use of program activities for the long-term achievement of desirable program outcomes [18]. Sustainability is a key element of CHW-led HIV services which are transitioning out of vertically funded sources [33–35].	

#### Area of harmonization: coordination

Coordination seeks to reduce duplication, fragmentation, confusion created by competing models, and overlap of responsibilities of differently trained CHWs, while building important synergies across CHW programs. This may be particularly productive in contexts with multiple faith-based, private, or other non-government CHW programs. A recent review of CHW-led ART programs in sub-Saharan Africa, for instance, identified at least six different CHW programs in Ethiopia, six in Malawi, and eight in Uganda [36]. In such settings, it is likely that each program has its own contracts and arrangements for health workers in their programs and parallel projects are funded, delivered, and reported separately [22]. Coordination among partners may reduce duplication and help identify synergies among programs possibly leading to overall efficiency gains. Working with and through existing local health services and mechanisms can help to strengthen them and avoids the creation of parallel HIV services and/or competitive working practices. Indeed, coordinated donor efforts can strengthen government efforts. In Rwanda, a non-governmental organization (NGO) provided coordinated salary support to government community health initiatives, strengthening the Ministry of Health’s community HIV program [37]. Coordinating the training and curriculum of CHWs with other health workforce also has educational benefits, since CHWs may benefit from interactions during their training with other aspiring health workers. Coordination may avoid differences in training/career prospects between programs, which may lead to friction across programs operated by different organizations [22]. Coordination, however, should also allow innovative experimentation with different approaches (as opposed to just imposing a uniform program).

##### Factors affecting coordination

Additional file 1: Table S2 displays key factors facilitating and inhibiting coordination of CHW programs. Facilitators of coordination include a clearly identified authority to oversee CHW programs [22]; solid government policy and a regulatory and organizational framework anchoring CHWs into the public or private health system; districts with strong planning and information systems; a common funding pool and plan; and appropriate supervision and support [38]. Sector-wide support platforms, for instance, can coordinate the actions of multiple entities in support of health system development and service delivery [38]. A few examples are the International Health Partnership (a group of national governments, development agencies, and civil society organizations, which promotes development cooperation in the health sector), the Human Resources for Health “Country Coordinating Mechanisms” (national committees that submit funding applications on behalf of the entire country to the Global Fund to obtain funding for HIV-related projects), and the National Human Resources for Health working groups involving a range of CHW stakeholders. Strategic collaborative partnership between communities and health systems also offers the potential for accelerating progress in improving CHW performance at scale [39]. On the other hand, barriers to coordination include, for instance, the “NGO challenge”: NGOs who compete with each other for resources, rather than working together to streamline the various CHW programs [38]. The incentive structure of this financing system compels NGOs to offer a different “product” than their competitors, which may not only provide positive innovations but also simply drive different approaches that are not effective [38].

#### Area of harmonization: integration

In this study, integration refers to the alignment of either donor-supported or national CHW programs with existing networks of larger health and/or community health systems. Integration can play an important role in further clarifying responsibilities, standardizing CHW programs, establishing accountability, and establishing career paths and professional associations [38]. In Kenya, for instance, integration of HIV services into primary care services was associated with significant increases in patient satisfaction [40]. In Brazil, the integration of CHWs into the existing civil service structure led to the compatibility of programs with government, financial, and service delivery health systems, whereas in India, integration reduced conflict between different actors of the health sector [41]. The integration of CHW services into national health systems or a large private provider can take different forms, such as partial integration (e.g., though not part of civil service, CHWs receive standardized incentives from the government, in the case of integration into national health systems) or full integration (e.g., CHWs that are part of the civil service and paid standardized monthly salaries by the government).

##### Factors affecting integration

Factors facilitating integration include the perceived relative advantage of nationally recognized CHW programs (Additional file 1: Table S2). In Ethiopia, CHW clients preferred to receive health-related information or advice from Health Extension Workers over other community volunteers [42]. Similarly, a positive perspective of CHWs by politicians and community members can facilitate integration. Community members reported positive perceptions of CHWs in Ethiopia and Pakistan [42, 43]. For CHW programs where women may play a more predominant role (such as those that have integrated HIV services with sexual and reproductive health services [32]), improved women’s rights and empowerment may also increase integration [44]. On the other hand, factors inhibiting integration of CHW programs may include a lack of proper definition of tasks of CHWs. In Brazil, a limited definition of CHW tasks affected acceptability of CHWs by other health workers, resulting in sub-optimal integration into the health system [45]. In Pakistan and India, discrimination based on social-cultural practices, age, and/or marital status has hindered the full integration of CHW programs [46, 47]. The existence of parallel and hierarchical communication structures may also affect integration. In India, a hierarchical communication structure resulted into rigidity and top-down power which constrained the flow of health information [48]. Other barriers include ineffective incentive structures of CHWs and inadequate infrastructure and supplies [41], in addition to lack of ownership and political support for CHW programs [22].

#### Area of harmonization: sustainability

Sustainability of the HIV response suggests that a country has the enabling environment, services, systems, and resources required to effectively and efficiently control the HIV epidemic in the long term [34]. Sustainability is a key challenge for CHW programs predominantly supported by uncertain and/or transitioning donor funding [30]. Researchers and policy-makers have increasingly sought to understand how the longer term sustainability of these programs can be better assured, thus enabling the continued and improved achievement of desirable program outcomes [18]. In addition to allowing systems to keep operating, the sustainability of a program could enable CHW initiatives to take a longer time horizon and better anticipate future needs (such as those resulting from changes in HIV treatment guidelines). Sustained programs may also be able to focus on investments whose returns are likely to be evident only after some time.

##### Factors affecting sustainability

Previous reviews on sustainability have suggested three important influences, including (i) program-specific factors (e.g., CHW program design and implementation), (ii) organizational factors and setting (e.g., program leadership), and (iii) underlying contextual factors [20, 31] (Additional file 1: Table S2). One factor facilitating the sustainability of CHW programs may be the program’s consistent and adequate supervision of CHWs. In South Africa, for instance, clinical leaders directly supervised CHW programs [49], whereas in India, supervision included biweekly on-site supervision of CHWs [50]. Another likely facilitating factor is the degree of “community fit” [18]. In Uganda, CHWs were selected by members of their community which likely reduced CHW attrition [51]. Other facilitating factors include the integration with the broader environment. In Botswana, CHWs worked alongside professional nurses in health facilities and received government pensions [52]. Conversely, factors inhibiting sustainability may include insufficient pay or incentives for CHWs relative to other employment opportunities [46]. The absence of sufficient financial incentives and availability of more lucrative employment elsewhere were causes of attrition among CHWs in the Democratic Republic of the Congo (DRC), Mozambique, and Nigeria [53–55]. Other factors inhibiting sustainability include a lack of community support for CHWs and distrust [56], in addition to limited human, technical, and financial resources. In the DRC, for instance, communities were dissatisfied that CHWs provided preventative services (as opposed to treatment services) [53].

### Analytic framework for harmonization of CHW programs

Atun et al. propose a framework to consider the diffusion of health sector initiatives into the broader health system [26]. First proposed as an approach to systematically consider the integration of health sector activities, expanding the lens to include other priority areas of harmonization may allow us to better understand why interventions may fail to achieve full harmonization, even in the face of many facilitating factors. A key advantage of extending this framework to other priority areas of harmonization (beyond integration alone) is that it suggests a common language that can also be applied to assess harmonization more broadly. We thus do not suggest a new framework, but rather a “framework +” that enables the systematic and holistic exploration of the extent to which different CHW interventions are harmonized in varied settings and the reasons for the variation. Specifically, in seeking to understand integration, Atun et al. argue for a broad approach, noting that “the extent to which [health sector interventions] are integrated…will be influenced by the nature of the problem being addressed, the intervention, the adoption system [stakeholders], the health system characteristics, and the broad context”.

**Fig. 1:**
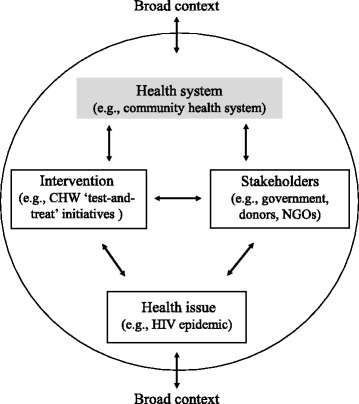
Framework for analyzing the harmonization of CHW programs for HIV

Each of these five elements of the analytic framework have applicability to harmonization activities for CHW programs for HIV and can be described as they contribute toward a harmonized approach for specific CHW interventions (Fig. 1). First, a *health priority* must be considered in light of other health priorities, and the urgency and scale of the issue, including the social narrative which surrounds it. More urgent issues, for instance, may initially necessitate a more targeted approach, with efforts for integration occurring further down the line. Second, less complex and better known CHW *interventions* may be easier to duplicate and likely to be more amenable to integration than newer or more complicated interventions which must be customized to specific target groups. CHW programs delivering HIV services, for instance, might not be considered as straightforward as childhood immunizations (i.e., an easily identifiable target group and schedule that makes it highly adaptable), but less complex than maternal and child health programs with multiple interrelated interventions rolled into one. Third, the perceptions and relative power of the various *stakeholders* involved with CHW programs is a critical question in the path to harmonization. The adoption and implementation of CHW programs often depends on a wide range of actors, including various government officials, community leaders, donors, and expert observers; and the presence of advocates can be a key determinant. Fourth, integration and sustainability further depend on the broader *health system*’s structural and financial capacity to absorb CHW programs. Finally, the *broader context*, including the “demographic, economic, political, legal, ecological, sociocultural…and technological factors in the environment” [26] can play a critical role in enabling or hindering CHW program harmonization. Populations in wealthier settings, for instance, may be more hesitant to see CHWs as “appropriate” health providers vis-à-vis professional health workers.

### Mapping three harmonization priority areas to analytic framework

Finally, we incorporate the three priority areas of harmonization into our extended analytic “framework +”. Table 2 considers different topics included in our review, categorized by the elements of our framework (the nature of the health issue, the intervention, the various stakeholders, health system characteristics, and broader context of CHW programs) and three priority areas of harmonization (coordination, integration, and sustainability). This five-by-three table allows us to visualize and better understand why CHW programs may fail to achieve either partial or full harmonization. It also allows us to test the assumptions that the topics listed in the table contribute to harmonization in future empirical studies. We note that there is significant overlap of topics across areas of harmonization and elements of the analytic framework. The perception of a CHW’s program effectiveness among community members and policy-makers, for instance, appears both in the integration and sustainability columns in Table 2. Both coordination and integration into the wider health system are also oft-cited facilitators of sustainability (e.g., for their contribution to be sustained, CHW programs may need to be integrated into the wider health system [57]). Harmonization spans many levels of the health system, and the three priority areas are deeply intertwined. Nevertheless, the various topics listed in Table 2 could be considered by policy-makers and researchers and addressed for a more harmonized approach to community-based health worker programs for HIV.

**Table 2:** Mapping priority areas of CHW program harmonization to analytic framework

	Coordination	Integration	Sustainability
Framework			
Health issue	Coordination between HIV and other health priorities; availability of a standardized community healthcare package	Variability in health priorities between national and sub-national levels	Easily identifiable health issues; broadness of focus and training of CHWs; reach of coverage of services
Intervention	Existence of cadres with specialized skills (which may be more complex to manage and evaluate); existence of parallel training and support structures for CHWs	Equivalence between differently trained CHWs; CHW hiring procedures; level of workload and supervision of CHWs; existence of standardized incentives; level of professionalization	Level of workload and supervision of CHWs; local modifiability; existence of standardized incentives; community participation and involvement of local decision-makers; CHW demographics; gender bias
Stakeholders	Number of stakeholders; awareness of need for coordination; existence of similar funding timelines, forums (such as working groups) and reports; result-oriented programming and reporting; “NGO challenge”	Perceived effectiveness of program; involvement of multiple public or private actors; position and power of health professionals; pace of CHW scale-up; dependence on external actors	Strength of leadership; level of commitment to coordination and integration; dependence on external actors; perceived effectiveness of the intervention; level of community buy-in
Health system	Existence of a single organizational structure dedicated to community health initiatives; level of decentralization; training of health workers; coordination with health facilities	Formal recognition of CHW programs by government; parallel supply chains; standardized training, supervision and monitoring of CHWs; public or private capacity; existence of a common funding pool	Public or private resources; existence of CHW training refreshers; attrition among young CHWs; coordination and integration of CHW programs; “NGO challenge”; predictability of funding
Broad context	Level of political support among all stakeholders and across government levels (or large private providers) for CHW-led services	Level of CHW program compatibility with local (community) structures; socioeconomic context and cultural values; political support for CHWs; community perception	Level of alignment with community norms and needs; level of political support and economic growth; level of support from external actors

*CHW* community health worker, *NGO* non-governmental organization, *MOH* Ministry of Health

## Discussion

This narrative review of the published and gray literature further defines the concept of harmonization, introduces three priority areas, provides an overview of factors thought to facilitate or hinder each, and integrates them into an analytic framework. While the three priority areas and elements of the analytic framework are interconnected and the overlapping drivers of these concepts complicate the establishment of concise definitions, each acts in a distinct way, and each faces its unique challenges. Factors facilitating and inhibiting harmonization are also highly context-specific, and increased harmonization is likely to be a complex political process, with in many cases incremental steps toward improvement. In settings with decentralized government, for instance, minor steps may be required to achieve harmonization of CHW programs for HIV. Conversely, countries with a stronger central government or a large existing national CHW program may be able to achieve harmonization rather quickly. The government of Rwanda, for instance, coordinated salary support to CHWs with a non-governmental organization [37], and Brazil and Ethiopia placed their CHWs entirely into an existing civil service structure [41], which substantially facilitated the harmonization of their CHW programs. One advantage of the conceptual approach suggested in this study is that it proposes a common language and framework that can be applied across different settings. This framework is likely informative to country-level decision-makers in settings with a large HIV epidemic, complex health systems, and multiple donors, in addition to other stakeholders involved with community health initiatives in low- and middle-income settings.

The analytic framework can be applied, for instance, in countries to explore which factors may be particularly important for increased harmonization in order to inform policy and practice in ways that can lead to improved health system performance. While the expansion of CHWs and their roles remains an explicit strategy in global efforts to end the HIV epidemic [9, 13], there are few documented cases of integrating donor-led HIV CHW programs into larger providers and sustaining HIV service delivery in the long term. Future research should also carefully map harmonization efforts at the sub-national and community level, where many CHW programs are implemented (and should likely be coordinated). We also note that, while the literature generally highlights the benefits of harmonization, there is less discussion surrounding the trade-offs associated with it. It is not clear that the solution to the many difficulties associated with fragmented CHW programs lies in integrating them entirely into a national health system. The loose processes in the employment of CHWs, for instance, may have advantages [19]. The open-ended and dynamic nature of community structures may allow for innovations which improve inclusiveness, local flexibility, and a range of motivations to be part of a health system “continuum” [19].

Finally, we recently applied the analytical framework proposed in this article in a four-country study in Southern Africa to assess the harmonization of CHW-led HIV services (De Neve JW, Garrison-Desany H, Andrews KA, Sharara N, Boudreaux C, Gill R, Geldsetzer P, Vaikath M, Bärnighausen T, Bossert TJ: Harmonization of community health worker programs for HIV: a four-country qualitative study in Southern Africa, submitted). The analytical framework outlined here has facilitated analysis in systematically comparing and contrasting CHW-led HIV services across these four countries and to generate meaningful evidence and inform policy around the harmonization of CHW-led HIV services.

### Limitations

This study has a number of limitations. First, we note that this review is not a systematic review of harmonization of CHW programs and that we have sampled only a very small portion of the approximately 38 000 titles returned by PubMed and Google Scholar with harmonization and community health worker in their texts. Our intention was neither a comprehensive review of all the available evidence nor an evaluation of the scientific quality of the articles but a narrative review to establish a clear definition of harmonization and a framework for analyzing the harmonization of CHW programs focused on CHW-led HIV services. Second, the definition of CHWs varies [58, 59]. While the lack of agreement upon a definition of CHWs is an important challenge in synthesizing evidence, we employed a broad definition [60]. Third, the distinction between CHWs who deliver HIV services and those who do not is not always clear in the literature. When evidence on CHWs delivering HIV services specifically was scarce, we supplemented our review with evidence on CHW programs that offered related health services.

## Conclusions

To our knowledge, this study is among the first to provide guidance for future research and policy to understand the process by which harmonization of CHW programs for HIV might be achieved. This study further defines harmonization, proposes three priority areas (coordination, integration, and sustainability), identifies a set of factors likely to facilitate or inhibit each, and suggests a framework which permits a systematic assessment of existing CHW programs.

## Additional file

Additional file 1: Additional context and research gaps. (DOCX 68 kb)

## References

[1] Perry HB, Zulliger R, Rogers MM (2014). Community health workers in low-, middle-, and high-income countries: an overview of their history, recent evolution, and current effectiveness. Annu Rev Public Health.

[2] World Health Organization (2008). Task shifting. Rational redistribution of tasks among health workforce teams: global recommendations and guidelines.

[3] Callaghan M, Ford N, Schneider H. A systematic review of task-shifting for HIV treatment and care in Africa. Hum Resour Health. 2010;8:8. doi: 10.1186/1478-4491-8-8 10.1186/1478-4491-8-8 PMC2873343 20356363

[4] Barr D, Odetoyinbo M, Mworeko L, Greenberg J (2015). The leadership of communities in HIV service delivery. Aids.

[5] Farmer P, Léandre F, Mukherjee JS, Claude MS, Nevil P, Smith-Fawzi MC, Koenig SP, Castro A, Becerra MC, Sachs J (2001). Community-based approaches to HIV treatment in resource-poor settings. Lancet.

[6] Kisesa A, Chamla D (2016). Getting to 90–90–90 targets for children and adolescents HIV in low and concentrated epidemics. Curr Opin HIV AIDS.

[7] Lewin S, Munabi-Babigumira S, Glenton C, Daniels K, Bosch-Capblanch X, van Wyk BE, Odgaard-Jensen J, Johansen M, Aja GN, Zwarenstein M, Scheel IB. Lay health workers in primary and community health care for maternal and child health and the management of infectious diseases. Cochrane Database Syst Rev. 2010, Issue 3. Art. No.: CD004015. doi:10.1002/14651858.CD004015.pub3. 10.1002/14651858.CD004015.pub3 PMC6485809 20238326

[8] Nachega JB, Adetokunboh O, Uthman OA, Knowlton AW, Altice FL, Schechter M, Galárraga O, Geng E, Peltzer K, Chang LW (2016). Community-based interventions to improve and sustain antiretroviral therapy adherence, retention in HIV care and clinical outcomes in low- and middle-income countries for achieving the UNAIDS 90-90-90 targets. Curr HIV/AIDS Rep.

[9] UNAIDS (2015). The critical role of communities in reaching global targets to end the AIDS epidemic.

[10] Grimsrud A, Bygrave H, Doherty M, Ehrenkranz P, Ellman T, Ferris R, Ford N, Killingo B, Mabote L, Mansell T, et al. Reimagining HIV service delivery: the role of differentiated care from prevention to suppression. J Int AIDS Soc. 2016;19:21484. doi:10.7448/IAS.19.1.21484. 10.7448/IAS.19.1.21484 PMC5136137 27914186

[11] Kober K, Van Damme W (2004). Scaling up access to antiretroviral treatment in southern Africa: who will do the job?. Lancet.

[12] Singh P, Sachs JD (2013). 1 million community health workers in sub-Saharan Africa by 2015. Lancet.

[13] UNAIDS. Plan to increase community health workers endorsed. 2017**:** http://www.unaids.org/en/resources/presscentre/featurestories/2017/february/20170213_community-health-workers.

[14] Perry H, Crigler L, Lewin S, Glenton C, LeBan K, Hodgins S. A new resource for developing and strengthening large-scale community health worker programs. Hum Resour Health. 2017;15:13. doi:10.1186/s12960-016-0178-8. 10.1186/s12960-016-0178-8 PMC5301425 28183351

[15] Schneider H, Lehmann U (2016). From community health workers to community health systems: time to widen the horizon?. Health Systems & Reform.

[16] Lehmann U, Van Damme W, Barten F, Sanders D (2009). Task shifting: the answer to the human resources crisis in Africa?. Hum Resour Health.

[17] Naimoli JF, Frymus D, Quain E, Roseman E (2012). Community and formal health system support for enhanced community health worker performance.

[18] Pallas SW, Minhas D, Perez-Escamilla R, Taylor L, Curry L, Bradley EH (2013). Community health workers in low- and middle-income countries: what do we know about scaling up and sustainability?. Am J Public Health.

[19] Schneider H, Hlophe H, van Rensburg D (2008). Community health workers and the response to HIV/AIDS in South Africa: tensions and prospects. Health Policy Plan.

[20] Shediac-Rizkallah MC, Bone LR (1998). Planning for the sustainability of community-based health programs: conceptual frameworks and future directions for research, practice and policy. Health Educ Res.

[21] Global Health Workforce Alliance (2010). Integrating community health workers in national health workforce plans.

[22] Mogedal S, Wynd S, Afzal MM (2013). Community health workers and universal health coverage: a framework for partners’ harmonized support.

[23] UNAIDS (2004). “Three Ones” key principles. Coordination of national responses to HIV/AIDS: guiding principles for national authorities and their partners.

[24] Walker PR, Downey S, Crigler L, LeBan K (2013). CHW “principles of practice” guiding principles for non-governmental organizations and their partners for coordinated national scale-up of community health worker programmes.

[25] Frymus D, Kok M, de Koning K, Quain E (2013). Knowledge gaps and a need based Global Research Agenda by 2015.

[26] Atun R, de Jongh T, Secci F, Ohiri K, Adeyi O. Integration of targeted health interventions into health systems: a conceptual framework for analysis. Health Policy Plan. 2010;25:104–11. 10.1093/heapol/czp055 19917651

[27] Gruen RL, Elliott JH, Nolan ML, Lawton PD, Parkhill A, McLaren CJ, Lavis JN (2008). Sustainability science: an integrated approach for health-programme planning. Lancet.

[28] The Altarum Institute. Defining sustainability of federal programs based on the experiences of the Department of Health and Human Services Office on Women’s Health multidisciplinary health models for women. 2009: https://www.womenshealth.gov/files/assets/docs/federal-reports/sustainabilityreview-060109.pdf

[29] Scheirer MA (2005). Is sustainability possible? a review and commentary on empirical studies of program sustainability. Am J Eval.

[30] Bamberger M, Cheema S (1990). Case studies of project sustainability: implications for policy and operations from Asian experience.

[31] Bossert TJ (1990). Can they get along without us? Sustainability of donor-supported health projects in Central America and Africa. Soc Sci Med.

[32] Hope R, Kendall T, Langer A, Bärnighausen T (2014). Health systems integration of sexual and reproductive health and HIV services in Sub-Saharan Africa. JAIDS Journal of Acquired Immune Deficiency Syndromes.

[33] PEPFAR. Human resources for health strategy. 2015: https://www.pepfar.gov/documents/organization/237389.pdf

[34] PEPFAR. Sustainable HIV epidemic control, PEPFAR Position Paper. 2016: https://www.pepfar.gov/documents/organization/264884.pdf

[35] The Global Fund. Sustainability, transition and co-financing Policy, Global Fund 35th Board Meeting. 2016: https://www.theglobalfund.org/media/4221/bm35_04-sustainabilitytransitionandcofinancing_policy_en.pdf

[36] Hermann K, Van Damme W, Pariyo GW, Schouten E, Assefa Y, Cirera A, Massavon W (2009). Community health workers for ART in sub-Saharan Africa: learning from experience—capitalizing on new opportunities. Hum Resour Health.

[37] Rich ML, Miller AC, Niyigena P, Franke MF, Niyonzima JB, Socci A, Drobac PC, Hakizamungu M, Mayfield A, Ruhayisha R (2012). Excellent clinical outcomes and high retention in care among adults in a community-based HIV treatment program in rural Rwanda. J Acquir Immune Defic Syndr.

[38] Tulenko K, Møgedal S, Afzal MM, Frymus D, Oshin A, Pate M, Quain E, Pinel A, Wynd S, Zodpey S (2013). Community health workers for universal health-care coverage: from fragmentation to synergy. Bull World Health Organ.

[39] Naimoli JF, Perry HB, Townsend JW, Frymus DE, McCaffery JA. Strategic partnering to improve community health worker programming and performance: features of a community-health system integrated approach. Hum Resour Health. 2015;13:46. doi:10.1186/s12960-015-0041-3. 10.1186/s12960-015-0041-3 PMC4556219 26323276

[40] Odeny TA, Penner J, Lewis-Kulzer J, Leslie HH, Shade SB, Adero W, Kioko J, Cohen CR, Bukusi EA (2013). Integration of HIV care with primary health care services: effect on patient satisfaction and stigma in Rural Kenya. AIDS Research and Treatment.

[41] Zulu J, Kinsman J, Michelo C, Hurtig A-K (2014). Integrating national community-based health worker programmes into health systems: a systematic review identifying lessons learned from low-and middle-income countries. BMC Public Health.

[42] Birhanu Z, Godesso A, Kebede Y, Gerbaba M (2013). Mothers’ experiences and satisfactions with health extension program in Jimma zone, Ethiopia: a cross sectional study. BMC Health Serv Res.

[43] Afsar HA, Qureshi AF, Younus M, Gulb A, Mahmood A (2003). Factors affecting unsuccessful referral by the lady health workers in Karachi, Pakistan. J Pak Med Assoc.

[44] Balabanova D, Mills A, Conteh L, Akkazieva B, Banteyerga H, Dash U, Gilson L, Harmer A, Ibraimova A, Islam Z (2013). Good health at low cost 25 years on: lessons for the future of health systems strengthening. Lancet.

[45] Zanchetta MS, McCrae Vander Voet S, Galhego-Garcia W, Smolentzov VM, Talbot Y, Riutort M, Galhego AM, de Souza TJ, Caldas RS, Costa E, et al. Effectiveness of community health agents’ actions in situations of social vulnerability. Health Educ Res. 2008;24:330–42. 10.1093/her/cyn023 18477582

[46] Kumar S, Kaushik A, Kansal S (2012). Factors influencing the work performance of ASHA under NRHM—a cross sectional study from eastern Uttar Pradesh. Indian Journal of Community Health.

[47] Wazir MS, Shaikh BT, Ahmed A (2013). National program for family planning and primary health care Pakistan: a SWOT analysis. Reprod Health.

[48] Scott K, Shanker S (2010). Tying their hands? Institutional obstacles to the success of the ASHA community health worker programme in rural north India. AIDS Care.

[49] van Ginneken N, Lewin S, Berridge V (2010). The emergence of community health worker programmes in the late apartheid era in South Africa: an historical analysis. Soc Sci Med.

[50] Bang AT, Bang RA, Reddy HM (2005). Home-based neonatal care: summary and applications of the field trial in rural Gadchiroli, India (1993 to 2003). J Perinatol.

[51] Katabarwa MN, Habomugisha P, Richards FO, Hopkins D (2005). Community-directed interventions strategy enhances efficient and effective integration of health care delivery and development activities in rural disadvantaged communities of Uganda. Trop Med Int Health.

[52] Gilson L, Walt G, Heggenhougen K, Owuor-Omondi L, Perera M, Ross D, Salazar L (1989). National community health worker programs: how can they be strengthened?. J Public Health Policy.

[53] Delacollette C, Van der Stuyft P, Molima K (1996). Using community health workers for malaria control: experience in Zaire. Bull World Health Organ.

[54] Emukah EC, Enyinnaya U, Olaniran NS, Akpan EA, Hopkins DR, Miri ES, Amazigo U, Okoronkwo C, Stanley A, Rakers L (2008). Factors affecting the attrition of community-directed distributors of ivermectin, in an onchocerciasis-control programme in the Imo and Abia states of south-eastern Nigeria. Ann Trop Med Parasitol.

[55] Simon S, Chu K, Frieden M, Candrinho B, Ford N, Schneider H, Biot M (2009). An integrated approach of community health worker support for HIV/AIDS and TB care in Angónia district, Mozambique. BMC International Health and Human Rights.

[56] Geldsetzer P, Vaikath M, De Neve J-W, Bossert TJ, Sibandze S, Mkhwanazi M, Bärnighausen T. Distrusting community health workers with confidential health information: a convergent mixed-methods study in Swaziland. Health Policy Plan. 2017;32(6):882-889. 10.1093/heapol/czx036 28407083

[57] Mwai GW, Mburu G, Torpey K, Frost P, Ford N, Seeley J (2013). Role and outcomes of community health workers in HIV care in sub-Saharan Africa: a systematic review. J Int AIDS Soc.

[58] Campbell J, Admasu K, Soucat A, Tlou S (2015). Maximizing the impact of community-based practitioners in the quest for universal health coverage. Bull World Health Organ.

[59] World Health Organization (2016). WHO guidelines on health policy and system support to optimize community health worker programmes.

[60] Lees S, Kielmann K, Cataldo F, Gitau-Mburu D (2012). Understanding the linkages between informal and formal care for people living with HIV in sub-Saharan Africa. Glob Public Health.

[61] Buse K, Walt G (1996). Aid coordination for health sector reform: a conceptual framework for analysis and assessment. Health Policy.

