# Supplementary material for: Harmonizing community-based health worker programs for HIV: a narrative review and analytic framework
Source: Hum Resour Health. 2017 Jul 3;15:45. doi: 10.1186/s12960-017-0219-y (PMC5496353; doi:10.1186/s12960-017-0219-y)
Supplement: Additional file 1: — Additional context and research gaps. (DOCX 68 kb) [file 12960_2017_219_MOESM1_ESM.docx]

**Additional files**

In this supporting materials appendix to the manuscript, “Harmonizing community-based health worker programs for HIV: a narrative review and analytic framework”, we provide:

- Box S1: definition of CHWs
- Additional background on current evidence and research gaps
- Supplementary tables S1 – S2

**Box S1: Definition of CHWs**

We define a CHW as “any health worker who performs functions related to health care delivery; has trained in some way in the context of the intervention; and has no formal professional or paraprofessional certificate or degree in tertiary education” [1]. The definition of CHWs varies and may include cadres that straddle the facility and the community (as opposed to only the community) [2, 3]. While a generalized lack of agreement upon definitions of CHWs is an important challenge in synthesizing evidence on the cadre, a recent review suggests some universal features. These include limited training, diverse typologies and titles, which may or may not be directly related to their roles, and their primary focus on communities [4]. Efforts to standardize definitions and typologies are ongoing [5, 6].

**Additional background on current evidence and research gaps**

Much of the evidence on CHWs focuses on the impact of particular programs on targeted health outcomes, including HIV and TB care, and maternal and child health service utilization. Studies have shown mixed results. A recent Cochrane review assessed evidence of effectiveness across a range of interventions. The delegation of HIV tasks to CHWs in Brazil, Ethiopia, Malawi, Namibia, and Uganda increased access to HIV services, particularly in rural areas and among underserved communities, and improved the quality of care [7]. CHWs also improved TB cure and child immunization rates, but the study found little evidence that CHWs increased TB preventive treatment completion or affected care seeking for childhood illness [1]^[[1]](#footnote-1)^. The large number and diversity of CHW programs may help to understand some of these mixed findings. Community-based responses to health challenges have evolved into multiple, stand-alone CHW programs with varying degrees of support and reporting [8]. Some of this diversity has been linked to the funding systems rather than need as, within countries and within sectors, some actors may prefer to work in silos as organizations and/or seek to attract donor funds by distinguishing themselves [9]. At the same time, there has often been a need for more increased human resources quickly, which results in the need to create new cadres (such as in the emergency phase in PEPFAR) [10]. Not only do the programmatic objectives differ, but so, too, do the support structures.

The emphasis in the development sector and human resources for health community is increasingly shifting from a focus on the impact of CHW-led interventions to the systems requirements for implementing and sustaining CHW programs at scale (i.e., the ‘community health system’ [11]). A recent review on research gaps commissioned by the GHWA, for instance, identified a number of questions regarding the appropriate design of CHW programs. The authors find little evidence to suggest which roles are best suited for the cadre, and the optimal structure of the CHW position itself, including the selection, training, supervision, and payment of CHWs remains unclear. The biggest gaps identified are around support interventions for supporting CHW performance and using more appropriate research methods [12]. Additionally, while the local development of CHW programs has been lauded for its responsiveness to local needs and capacity to innovate, the work of the GHWA also represents a growing consensus that the benefits of increased coordination outweigh its challenges and/or disadvantages [13]. Fragmentation, duplication of services, and weak connections with the formal health system may further undermine CHW commitment and ability to achieve sectorial goals [13]. Despite increasing attention to these challenges, there are currently few systematic efforts to improve our understanding of the process by which harmonization might be achieved, and the path to integration of CHW programs into the national health system remains unclear. In Table S1, we show outstanding research gaps surrounding harmonization that were identified in the recent literature.

Table S1: Research gaps related to CHW program harmonization, by priority area

CHW: community health worker. See references for full citation information: [1, 2, 9, 14-20].

Table S2: Factors facilitating and inhibiting harmonization, by priority area

CHW: community health worker.

**References for appendix**

1. Lewin S, Munabi-Babigumira S, Glenton C, Daniels K, Bosch-Capblanch X, van Wyk BE, Odgaard-Jensen J, Johansen M, Aja GN, Zwarenstein M, Scheel IB: **Lay health workers in primary and community health care for maternal and child health and the management of infectious diseases.** *Cochrane Database Syst Rev* 2010**:**CD004015. doi: 10.1002/14651858.CD004015.pub3

2. Naimoli JF, Frymus D, Quain E, Roseman E: **Community and Formal Health System Support for Enhanced Community Health Worker Performance.** *A US Government Evidence Summit* 2012: https://www.usaid.gov/sites/default/files/documents/1864/CHW-Evidence-Summit-Final-Report.pdf

3. Naimoli JF, Perry HB, Townsend JW, Frymus DE, McCaffery JA: **Strategic partnering to improve community health worker programming and performance: features of a community-health system integrated approach.** *Human Resources for Health* 2015, **13**.doi: 10.1186/s12960-015-0041-3

4. Lees S, Kielmann K, Cataldo F, Gitau-Mburu D: **Understanding the linkages between informal and formal care for people living with HIV in sub-Saharan Africa.** *Glob Public Health* 2012, **7:**1109-1119.

5. Campbell J, Admasu K, Soucat A, Tlou S: **Maximizing the impact of community-based practitioners in the quest for universal health coverage.** *Bulletin of the World Health Organization* 2015, **93:**590-590A.

6. World Health Organization: **WHO guidelines on health policy and system support to optimize community health worker programmes.** *Guideline Development Group* 2016.

7. Celletti F, Wright A, Palen J, Frehywot S, Markus A, Greenberg A, de Aguiar RA, Campos F, Buch E, Samb B: **Can the deployment of community health workers for the delivery of HIV services represent an effective and sustainable response to health workforce shortages? Results of a multicountry study.** *AIDS* 2010, **24 Suppl 1:**S45-57.

8. Hermann K, Van Damme W, Pariyo GW, Schouten E, Assefa Y, Cirera A, Massavon W: **Community health workers for ART in sub-Saharan Africa: learning from experience – capitalizing on new opportunities.** *Human Resources for Health* 2009, **7:**31.

9. Tulenko K, Møgedal S, Afzal MM, Frymus D, Oshin A, Pate M, Quain E, Pinel A, Wynd S, Zodpey S: **Community health workers for universal health-care coverage: from fragmentation to synergy.** *Bulletin of the World Health Organization* 2013, **91:**847-852.

10. PEPFAR: **Human Resources for Health Strategy.** 2015: https://www.pepfar.gov/documents/organization/237389.pdf

11. Schneider H, Lehmann U: **From Community Health Workers to Community Health Systems: Time to Widen the Horizon?** *Health Systems & Reform* 2016, **2:**112-118.

12. Frymus D, Kok M, de Koning K, Quain E: **Knowledge gaps and a need based Global Research Agenda by 2015.** *Global Health Work Alliance Report* 2013.

13. Mogedal S, Wynd S, Afzal MM: **Community Health Workers and Universal Health Coverage: A Framework for Partners’ Harmonized Support.** *Global Health Workforce Alliance Report* 2013.

14. Glenton C, Colvin CJ, Carlsen B, Swartz A, Lewin S, Noyes J, Rashidian A, Glenton C: **Barriers and facilitators to the implementation of lay health worker programmes to improve access to maternal and child health: qualitative evidence synthesis.** 2013.

15. Haines A, Sanders D, Lehmann U, Rowe AK, Lawn JE, Jan S, Walker DG, Bhutta Z: **Achieving child survival goals: potential contribution of community health workers.** *The Lancet* 2007, **369:**2121-2131.

16. Lehman U, Sanders D: **Community health workers: what do we know about them? The state of evidence on programmes, activities, costs, and impact on health outcomes of using community health workers.** *WHO, Geneva Evidence and Information for Policy Department of Human Resources for Health* 2007.

17. Mwai GW, Mburu G, Torpey K, Frost P, Ford N, Seeley J: **Role and outcomes of community health workers in HIV care in sub-Saharan Africa: a systematic review.** *J Int AIDS Soc* 2013, **16:**18586.

18. Prasad MB, Muraleedharan V: **Community Health Workers: A Review of Concepts, Practice and Policy Concerns.** *The HRH Global Resource Center* 2007.

19. Schneider H, Hlophe H, van Rensburg D: **Community health workers and the response to HIV/AIDS in South Africa: tensions and prospects.** *Health Policy and Planning* 2008, **23:**179-187.

20. Zulu J, Kinsman J, Michelo C, Hurtig A-K: **Integrating national community-based health worker programmes into health systems: a systematic review identifying lessons learned from low-and middle-income countries.** *BMC Public Health* 2014, **14:**987.

1. The absence of evidence in Cochrane reviews may be a function of inadequate research methods (since Cochrane standards for research are demanding) and should not necessarily be taken as definitive rejection of relationships. [↑](#footnote-ref-1)
